# Subclinical inflammation in a case of menstruation-induced familial Mediterranean fever: A case report

**DOI:** 10.1097/MD.0000000000012305

**Published:** 2018-09-21

**Authors:** Kazusato Hara, Yushiro Endo, Midori Ishida, Yuya Fujita, Sosuke Tsuji, Ayuko Takatani, Toshimasa Shimizu, Remi Sumiyoshi, Takashi Igawa, Masataka Umeda, Shoichi Fukui, Ayako Nishino, Shin-ya Kawashiri, Naoki Iwamoto, Kunihiro Ichinose, Mami Tamai, Hideki Nakamura, Tomoki Origuchi, Kiyoshi Migita, Atsushi Kawakami, Tomohiro Koga

**Affiliations:** a Department of Immunology and Rheumatology, Unit of Advanced Preventive Medical Sciences, Nagasaki University Graduate School of Biomedical Sciences, Nagasaki; b Department of Rheumatology, Fukushima Medical University School of Medicine, Fukushima, Japan.

**Keywords:** familial Mediterranean fever, *MEFV* gene, menstruation-induced familial Mediterranean fever, subclinical inflammation

## Abstract

**Rationale:**

Because most patients with familial Mediterranean fever (FMF) have attacks without any prodromal symptoms, and since it is suggested that patients with FMF have subclinical inflammation even during remission, a daily continuous administration of colchicine is recommended for patients with FMF even during remission. However, it is possible that intermittent colchicine therapy only during FMF attacks prevents the attacks completely in patients with FMF with expectable attacks.

**Patient concerns:**

A 31-year-old Japanese woman suffered high fever and arthralgia lasting for 2 to 3 days after each menstrual period's start. She was admitted to our hospital, and colchicine was administered immediately after her next period's start, and the febrile attack was completely prevented.

**Diagnoses:**

We eventually diagnosed typical FMF.

**Interventions:**

Her remission has been maintained by intermittent colchicine therapy.

**Outcomes:**

The genetic analysis revealed the G304R heterozygous mutation in exon 2 of the *MEFV* gene. Cytokine analysis suggested subclinical inflammation during the remission period.

**Lessons:**

This case suggests that taking an extensive medical history (including the relationship between fever attack and menstruation) is important in the diagnosis of female patients with FMF. This case also suggests that a continuous administration of colchicine may have to be considered to regulate subclinical inflammation even in patients with FMF with completely expectable attacks.

## 1 Introduction

Familial Mediterranean fever (FMF) is an inherited autoinflammatory disease caused by mutations of *Mediterranean fever* (*MEFV*) gene, which codes for a protein named pyrin.^[1]^ Patients with FMF have been commonly observed in the Mediterranean region,^[2]^ but patients with FMF have also been reported recently in Japan, where they have different genetic characteristics from the endemic areas.^[3]^ Nongenetic factors are also important in the etiologies of FMF, and it has been suggested that FMF attacks are preceded by several factors including menstruation.^[4]^ Although menstruation-induced FMF cases are not rare in the endemic areas,^[2]^ little has been reported on such cases in Japan.

Because most patients with FMF have attacks without any prodromal symptoms, and since it is suggested that patients with FMF have subclinical inflammations even during remission,^[5]^ a daily continuous administration of colchicine is recommended for patients with FMF even during remission.^[6]^ It is possible that intermittent colchicine therapy only during FMF attacks prevents the attacks completely, but this concept is limited to patients with FMF with completely expectable attacks.^[7,8]^ There is no consensus on intermittent colchicine therapy in any FMF cases.

We herein report the case of a woman with menstruation-induced FMF who was revealed to have a heterozygous mutation of exon 2 in *MEFV* gene, and whose FMF attacks were completely eliminated by intermittent colchicine therapy. The results of the cytokine analysis in her case suggested subclinical inflammations during the remission period.

## 2 Case report

The healthy 31-year-old Japanese woman had suffered from fever (≥38°C) that had lasted for 2 to 3 days along with arthralgia since May 2017. These recurrent attacks completely coincided with the start of her menstrual periods. In September 2017, she presented with a high fever and arthralgia that had occurred at the initiation of her menstruation. The fever attack disappeared once approximately 48 hours after the onset of fever, but the next day she presented a high fever again and was admitted to our department for the evaluation of fever of unknown origin.

On admission, her body temperature was 36.8°C, her blood pressure was 97/68 mm Hg, the heart rate was 97 beats/min, and the pulse oximetric saturation (SpO_2_) was 98% (room air). On physical examination, she had mild arthralgia without heat and swelling at the joints of both shoulders, elbows, and knees. She had no symptoms suggesting peritonitis or pleuritis, or erysipelas-like skin lesions.

Laboratory investigations showed the following results: white blood cell count 3600/μL (neutrophils 72.8%, lymphocytes 18.9%), hemoglobin (Hb) 12.0 g/dL, platelets 18.6×10^4^/μL, C-reactive protein (CRP) 9.53 mg/dL, ferritin 51 ng/mL (normal range 6.0–138 ng/mL), and serum amyloid A (SAA) 884.5 μg/mL. The serum complement level was normal. No abnormalities were revealed by a urinalysis, and no liver or renal dysfunction was detected.

The following immunologic and serologic results were all negative: rheumatoid factor, antinuclear antibody, proteinase-3 antineutrophil cytoplasmic autoantibodies (PR3-ANCAs), and myeloperoxidase antineutrophil cytoplasmic autoantibodies (MPO-ANCAs). The results of assays of β-d-glucan, T-SPOT.TB, and human parvovirus B19 were all negative. The results of assays of both cytomegalovirus and Epstein–Barr virus showed a pattern of her past infection.

A thoracico-abdominal enhanced computed tomography examination and gallium-67 (^67^G) scintigraphy revealed no abnormalities. She did not present a high fever after her admission to our department. Her arthralgia also disappeared spontaneously on the 1st hospital day, and the CRP level had decreased rapidly to the normal range. We suspected FMF based on the recurrent and self-limiting fever attacks, and we administered 1.0 mg/d of colchicine for 5 days immediately after the start of her next menstrual period. After this treatment, no further febrile attacks were observed. We finally diagnosed typical FMF on the basis of the Tel Hashomer criteria.^[9]^ A subsequent genetic analysis of exons 1, 2, 3, and 10 of the *MEFV* gene revealed the G304R heterozygous mutation in exon 2 of the *MEFV* gene.

**Figure 1:**
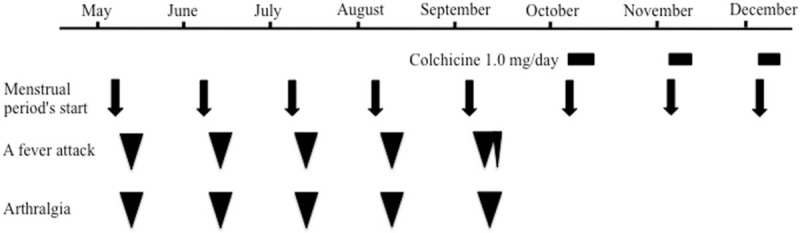
The clinical course of the patient, a 31-year-old Japanese woman. Black rectangles show intermittent colchicine therapy (1.0 mg/d of colchicine for 5 days at the start of each menstrual period). Black arrows show her menstrual period's start. Inverted triangles show her symptoms such as fever attacks and arthralgia.

We excluded other autoinflammatory diseases such as hyperimmunoglobulinemia D with periodic fever syndrome, cryopyrin-associated periodic syndrome, and tumor necrosis factor receptor-associated periodic syndrome based on the patient's medical history, physical findings, and laboratory findings. Her remission has been maintained by intermittent colchicine therapy (1.0 mg/d of colchicine for 5 days at the start of each menstrual period) for over 6 months as of this writing (Fig. 1).

We investigated the presence of subclinical inflammation in our patient's case by analyzing her serum cytokines at the attack and remission before the initiation of colchicine. Her levels of IL-1β, -4, -6, and -17 and TNF-α were higher than normal not only at the attack but also at the remission (Table 1). Although we recommended a daily continuous administration of colchicine to her to regulate subclinical inflammation, she refused to accept it because intermittent colchicine had improved her symptoms.

**Table 1:**
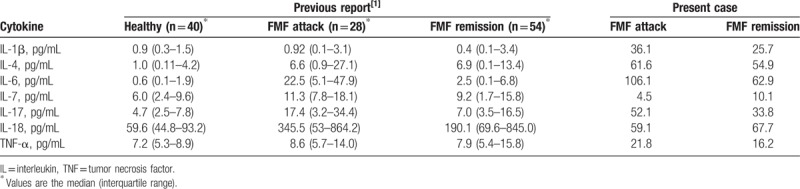
Comparison of cytokines in healthy controls, patients with FMF, and preset case during the attack and remission periods.

## 3 Discussion

We encountered a rare case of FMF, the symptoms of which appeared at the start of the patient's menstrual periods, treated successfully treated with an intermittent use of colchicine. FMF is an autoinflammatory disease characterized by recurrent attacks of fever with serositis, synovitis, and erysipelas-like skin lesions.^[10]^ FMF is the most prevalent in the Mediterranean and Middle Eastern regions, especially among Turks, Arabs, Armenians, and non-Ashkenazi Jews.^[2]^ However, an increasing number of FMF cases has been reported in countries outside of these regions, including Japan.^[11]^ The genetic characteristics of Japanese patients with FMF include a lower percentage of *MEFV* exon 10 mutations with high penetrance and a higher percentage of *MEFV* exon 2 mutations with low penetrance compared to Western countries.^[11]^ Most of the previously reported menstruation-induced patients with FMF had mutations in *MEFV* exon 10,^[2,7,12,13]^ but there has been no case report of a menstruation-induced patient with FMF with a mutation in only *MEFV* exon 2, to our knowledge. Although the precise mechanisms by which the exon 2 mutation affects the development of menstruation-induced FMF have not yet been elucidated, we speculate that our patient's case reflects the genetic characteristics of Japanese FMF.

The etiologies of FMF have not been completely elucidated. It was suggested that genetic factors other than the *MEFV* gene may be involved in FMF.^[14]^ A twin study of FMF showed variability in symptoms and severity within pairs of monozygotic twins, suggesting the importance of nongenetic factors in the clinical characteristics of FMF.^[15]^ FMF attacks might be preceded by several factors (eg, emotional or physical stress, exposure to cold, a high-fat meal, and menstruation).^[4]^ In a survey of 72 FMF female patients in an endemic area, 38 (53%) answered that their FMF attacks frequently coincided with their menstrual cycles.^[16]^ A study from Israel also revealed that 10 (7%) of 141 female patient with FMF had menstruation-induced FMF attacks. However, most of those patients were diagnosed with FMF before 10 years of age, and none had characteristics of menstruation-induced FMF attacks at the disease onset.^[2]^

Although menstruation-induced FMF is very rare in Japan compared to the endemic areas, Japanese menstruation-induced patients with FMF might be more likely to present menstruation-induced FMF attacks at disease onset. Both our patient and a previously reported patient with menstruation-induced FMF in Japan^[12]^ had adult-onset FMF, and they presented FMF attacks that were closely related to their menstruation cycles at the disease onset. Thus, it is very important for physicians to take careful medical histories that include an examination of the relationship between fever attacks and menstruation when female patients are suspected of having FMF.

It is suggested that sex hormones play important roles in the processes of inflammation and that hormonal alterations may result in the FMF attacks during menstruation. This hypothesis is supported by the findings that estrogen can inhibit interleukin (IL)-1β-induced IL-6 production^[17]^ and that the inhibition of IL-6 indirectly prevents FMF attacks.^[18]^ Estrogen has also been reported to mimic colchicine's effects on tubulin assembly and adhesion molecule expression.^[19,20]^ It is thus presumed that the lower level of estrogen during menstruation causes menstruation-induced FMF attacks.^[2]^ The following clinical observations also support our hypothesis: first, oral contraceptive drugs are effective for the prevention of menstruation-induced FMF attacks in some cases^[2,21]^; and second, many menstruation-induced patients with FMF have no FMF attacks during pregnancy, which involves a high level of estrogen.^[2,22]^

Treatment strategies for patients with FMF with completely expectable attacks such as those observed in menstruation-induced FMF have not been established. The erythrocyte sedimentation rate and the levels of CRP, fibrinogen, and leukocytes are useful biomarkers for predicting the acute-phase response during FMF attacks.^[23]^ These markers usually increase during the attacks and return to the normal range during remission.^[24]^ However, it was suggested that patients with FMF have subclinical inflammations even during remissions,^[5]^ and such sustainable inflammations may be accompanied by the risk of complications such as anemia, splenomegaly, lowered bone mineral density, heart disease, and amyloidosis, which is a fatal complication.^[24]^ For the purpose of preventing subclinical inflammation in patients with FMF, a daily continuous administration of colchicine is recommended even during remissions, and it is necessary to consider increasing the dose of colchicine at times of emotional and physical stress, as these can be triggers of FMF attacks.^[6]^

Physicians treating menstruation-induced patients with FMF were also advised to increase the patients’ dose of colchicine during menstruation in addition to the daily continuous administration of colchicine.^[2]^ It is also possible that intermittent colchicine therapy only during menstruation-induced FMF attacks prevents the attacks completely. A pair of reports noted that menstruation-induced patients with FMF were treated successfully with such intermittent colchicine therapy.^[7,8]^ Colchicine is a substrate of cytochrome P450 (CYP) 3A4 isoenzyme and P-glycoprotein-1 efflux transporter.^[25]^ Colchicine is an effective and safe drug for most patients with FMF, but it sometimes causes side effects (eg, convulsions, abdominal pain, hyperperistalsis, diarrhea, and vomiting).^[26]^ Importantly, an increase in the blood concentration of colchicine due to a concomitant medication causes severe pancytopenia and multiple organ failure, which can be fatal.^[27,28]^ Considering these side effects and the medication adherence, for patients with FMF with completely expectable attacks, it may be appropriate to introduce the use of intermittent colchicine therapy only at time points just before the attacks.

To the best of our knowledge, there have been no reports describing the presence of subclinical inflammation or the development of future complications in patients with FMF with completely expectable attacks such as our patient. The levels of serum IL-6 and tumor necrosis factor-alpha (TNF-α) were significantly higher in patients with FMF than in healthy controls during the attacks,^[29]^ as were the serum levels of IL-4, -7, -17, and -18.^[1]^ In other studies, the serum levels of IL-1β, -7, -17, and -18 were significantly higher in patients with FMF than in healthy controls even during the patients’ remissions, suggesting a contribution of these cytokines to subclinical inflammation.^[1,30]^ However, although the serum IL-18 level in patients with FMF with *MEFV* exon 10 mutations during the attacks and remissions was significantly higher than in healthy controls, there was no significant difference in the serum IL-18 level during the attacks and remissions between patients with FMF without *MEFV* exon 10 mutations and healthy controls.^[1]^

Our patient's levels of IL-1β, -4, -6, and -17 and TNF-α were higher than normal not only at the attack but also at the remission, suggesting subclinical inflammation. We speculate that normal range of serum IL-18 level in our patient's case reflects the characteristics of patients with FMF without MEFV exon 10 mutation, who have IL-18-independent inflammations. It is unknown how the subclinical inflammation in our patient's case may contribute to the development of long-term complications, but intermittent colchicine therapy only during menstruation-induced FMF attacks may fail to prevent subclinical inflammation during remissions. Thus, a continuous administration of colchicine may have to be considered even in patients with FMF with completely expectable attacks such as our patient. We hope that a further accumulation of reports of patients with FMF with completely expectable attacks and the analyses of serum cytokines at the attacks and remissions will enable a conclusion as to whether or not these patients need a daily continuous administration of colchicine.

In conclusion, we have described the case of a menstruation-induced patient with FMF with heterozygous mutation in *MEFV* exon 2. Further attacks have been completely prevented by intermittent colchicine therapy. Our patient's case suggests that taking an extensive medical history including the relationship between the patient's menstruation cycle and fever attacks is important in the diagnosis of FMF among female patients. Further studies are needed to clarify whether intermittent colchicine therapy for patients with FMF with expectable attacks can prevent FMF attacks completely and regulate subclinical inflammation, which can lead to organ damage.

## Author contributions

**Conceptualization:** Midori Ishida, Yuya Fujita, Sosuke Tsuji, Ayuko Takatani, Toshimasa Shimizu, Remi Sumiyoshi, Takashi Igawa, Masataka Umeda, Shoichi Fukui, Ayako Nishino, Shin-ya Kawashiri, Naoki Iwamoto, Kunihiro Ichinose, Mami Tamai, Hideki Nakamura, Tomoki Origuchi, Kiyoshi Migita.

**Supervision:** Atsushi Kawakami, Tomohiro Koga.

**Writing – original draft:** Yushiro Endo, Kazusato Hara.

**Writing – review & editing:** Yushiro Endo.
